# Chicago Medical Society, February 1st

**Published:** 1886-04

**Authors:** 


CHICAGO MEDICAL SOCIETY.

*Stated Meeting, February 1st, 1886.*—THE PRESIDENT, C. T. PARKES, M. D., in the chair.

DR. J. SUYDAM KNOX read a paper entitled

QUINSY AS A RHEUMATISM.

The author reported the treatment of fifty cases of the disease. In forty-nine cases (95 per cent.) there was a positive rheumatic diathesis. Forty of these cases recovered in thirty-six to seventy-two hours, without suppuration. The treatment was salicylate of sodium, and hot alkaline gargles, or the repeated insufflation on the tonsils of bicarbonate of soda. Five cases, decidedly rheumatic, were not benefited by similar treatment. Five cases, not rheumatic, were not benefited, and at the end of forty-eight hours the ordinary treatment of quinsy was followed. Only one of these cases did not suppurate. Doctor Knox concludes that in a large majority of cases quinsy is a rheumatic inflammation, for the following reasons: 1. From the percentage observed.

2. From the success of anti-rheumatic treatment. 3. From the similarity between the symptoms of quinsy and rheumatism.

DR. KNOX said that wherever possible he used an insufflator and blew bicarbonate of soda directly on the tonsils, using as much as 30 grains, and allowing it to remain as long as possible, to be followed by a gargle of water as hot as could be borne and to be continued until the throat was cleared, which would be from half an hour to two hours. He sometimes used carbolized lime water with morphia.

He stated that he could not say as to every case, but where suppuration takes place it is usually in the gland, an abscess is formed from glandular inflammation and suppuration takes place through the gland.

DR. A. B. STRONG reported

### A CASE OF INTUBATION OF THE LARYNX FOR ACUTE CATARRHAL LARYNGITIS, WITH RECOVERY.

The patient was a child, aged two and a half years, delicate and small for her age. She had been sick thirty-six hours when it was decided that intubation was imperatively demanded, and it was done. Instant relief was obtained. The act of introducing a No. 3 tube had caused such an abundant ejection of mucus, and the breathing was so easy, that the tube was withdrawn, and the child passed the night comfortably. However, at 4 P. M. of the succeeding day the tube was re-introduced with the same success, but about twelve hours after the child caught hold of the thread and withdrew the tube. The No. 2 tube was then placed in position and remained there sixty-eight hours, when recovery was complete. Dr. Strong said intubing the larynx has advantages over tracheotomy in being quickly performed, furnishing instant relief without cutting or bloodshed, being free from danger, and is readily assented to by the parents. The care of the patient after operation is slight, as compared with tracheotomy, as the tube does not have to be interfered with, nor does it often clog up. The tubes as now made, those of Dr. O'Dwyer, are not easily coughed out. It is easier to introduce than to remove the tube, owing to the fact that if the thread is left in the mouth it causes coughing and difficulty in swallowing. It was observed that during the thirteen hours the child wore the tube with the thread she had more difficulty in swallowing than subsequently when the thread was withdrawn; the thread being drawn across the epiglottis probably interferes with it closing the glottis during deglutition. Besides, the thread requires constant watching lest the child grasp it and withdraw the tube. Still in case a mass of mucus should lodge against the lower end of the tube and stop respiration, the thread might be the means of saving the child's life by allowing the tube to be speedily withdrawn.

THE PRESIDENT said it was a matter of surprise to him that so small an opening would allow the exit of secretions, as in the case just reported. It had been his experience that recovery always follows cases of catarrhal laryngitis. However, in these cases, he thought the introduction of the tube might be of great benefit in relieving difficult breathing or dyspnœa, and in allaying the fears of friends. He was aware that a very small opening gave air sufficient for inspiration for some hours. He once operated on a boy about 5 years old in whom the trachea by improper manipulation was turned in some way, and the incision made in the side of the trachea. When the external tracheotomy tube was introduced into the wound the child breathed quite well, the suffusion of the face passed away and the lips became red, but still the sound of the breathing was not satisfactory to the ear. He then tried to introduce the internal tube and found the child had a return of all the symptoms of suffocation. On removing the external tube at the bottom of the wound the trachea was seen unopened. The child recovered. The President said that while the tube was in this bad position he looked into it through the fenestra, and at the bottom of the fenestra was an opening through which the smallest probe could be introduced into the trachea, which enabled the child to inspire air enough to relieve the urgent symptoms. He thought that in a case of true diphtheritic laryngitis an opening of the size of the tube under discussion could not, in his opinion, give exit to any such amount of secretion as is frequently seen during an operation. He did not think that intubation of the larynx would take the place of tracheotomy; it no doubt is of great benefit in those cases where the patient is likely to die unless some measure be quickly adopted which will give time enough to allow the operation of tracheotomy. In the case under discussion the tube was worn sixty-eight hours continuously. He had not seen a case of tracheotomy where the closure of the fenestra gave evidence that the trouble with the larynx or glottis was overcome in less than six days. This was the shortest time in which he had been able to remove the tracheotomy tube.

PROFESSOR F. E. WAXHAM said that from his experience with intubation of the larynx he was thoroughly convinced of its utility, and its superiority over tracheotomy. He had eight recoveries out of his first seventeen cases, a result which he claimed could not be approched by tracheotomy, especially in Chicago. The ages of the patients varied from eleven months to five years; he considered these eight cases as being saved from certain death, as in only one case would tracheotomy have been permitted by the friends, and he had the corroborative evidence of other physicians as to the impending danger, and the urgent necessity of surgical interference. Since his last report he had had a number of cases, and had performed the operation four times during the last week, one patient being only eleven months old, suffering from both laryngeal and pharyngeal diphtheria; the urgent symptoms were at once relieved. In another case, age eighteen months, where death was impending, the tube was introduced without difficulty and the child relieved, and recovery would without doubt have been the result had not the child died of pneumonia on the second day. In another case, one of malignant diphtheria in a child of two years, the patient succumbed on the second day after the operation. In another case he found the patient cold and livid, pulseless, and unconscious. After the tube was introduced cold water was dashed on the child's face, and in about five minutes he looked around and asked for his father; took some milk and passed into a quiet sleep. This child died from pneumonia three hours later. Professor Waxham said that in the eight cases that recovered, in every instance false membrane was observed; when the tube was introduced the membrane was ejected, either in large flakes or broken-down masses. He recommended that in treatment after intubation nothing at all irritating should be given, as when a child takes fluid of any kind a few drops will trickle into the trachea and cause violent coughing, and this irritation will often lead to pneumonia. In a child rugged and strong bi-chloride of mercury may be given to hasten disintegration of false membrane. The most remarkable case which had come under his observation was a child of four years old that was on the verge of suffocation, when, upon the tube being introduced, a considerable portion of false membrane was thrown out through the tube and the violent symptoms subsided at once. The thread was removed, and on the second day after the operation the child was playing about the room and continued about the house during the four days that the tube was worn, and finally made a perfect recovery. Professor Waxham thought that in regard to the comparative value of tracheotomy and intubation very much might be said. The text-books give as the percentage of recoveries from tracheotomy about one in three, but these statistics are made up from the most favorable reports. If a physician has one recovery out of three or four cases he is justly proud of it and reports the case ; on the other hand, if there is one recovery out of fifteen or twenty cases, probably no report is made. He had known one physician to have operated fifty times with but two recoveries. He thought that the thread should always be removed, as it is a constant cause of irritation, and that no difficulty need be experienced in removing the tube with extractors. He thought intubation had a grand future.

PROFESSOR E. FLETCHER INGALS did not take an enthusiastic view of intubation, except for young children, when he thought it would be found more satisfactory than tracheotomy. In very young children tracheotomy does not result well, and he thought intubation would be unsatisfactory in older ones until we have larger tubes. He stated the accepted opinion of surgeons to be that a tube of less than one-fourth of an inch in diameter cannot furnish sufficient air for a child to live on. He thought that Dr. Waxham had been remarkably successful with intubation, and had demonstrated its utility, for which he deserved credit. He thought that intubation of the larynx is preferable to tracheotomy in children less than three and a half years of age; children much older than this cannot get a sufficient amount of air through the tube now in use. He said, also, that in performing one operation he had had trouble with the gag, which was not large enough for the child, a boy of five years, who lifted his teeth from the gag and closed them on the doctor's finger. He thought there was no need of the thread remaining, as there could be little difficulty in removing the tube. He thought, that in cases where it is difficult to get the consent of friends, or where the conditions are such that tracheotomy cannot be performed immediately, intubation would be of value; there are cases not membranous in which intubation may be of value. The statistics looked unfavorable for tracheotomy, but he had seen statistics of fifteen or sixteen cases where half of them were recoveries. His success had not been quite so good, but he attributed this mainly to the fact that he had operated on five children who were almost dead, or at least had stopped breathing before the operation began. He had the good fortune once to save a child who had not breathed for what seemed to him twenty minutes. One of the strong points in favor of intubation is that it may be done early, and it does no harm even if unnecessary.

DR. H. T. BYFORD said there was another way of drawing the line which would more accurately describe the usefulnes of the two operations. Intubation seemed to him the operation for private practice, and statistics so far are comparatively favorable to it as such. But the co-operation of the patient's friends, the preparation of the inspired air by passage through natural channels, the freedom of intubation from grave responsibility, its bloodlessness, the simplicity of treatment afterwards, as well as the greater rapidity with which the mucous membrane around the vocal cords will get well, are conditions which have less bearing in hospital practice, where we have trained nurses and all modern appliances, and there is more hope of success in tracheotomy. While he did not think this latter operation favorable for private practice, such advantages as having the tube under the eye, and within reach of the fingers of an attendant, the ease of local medication, the possibility of removing shreds of membrane and plugs of mucus, and of inspecting the parts by removing the tube and the longer time the tube can be retained, these are things that do not pertain to intubation, and which, in hospital practice, must secure for it some consideration. He said that there was one clinical fact that had not been mentioned in this connection, yet which, more than all other things put together, accounts for the success of intubation and the failure of tracheotomy as life-saving measures: in the one the patient can cough; in the other he cannot. After intubation the patient can normally close the glottis, compress the inclosed air in the lungs, and with sudden explosive force expel everything that is sufficiently loosened. This accounts for the fact that with such a small tube the patient experiences no difficulty. After tracheotomy the patient has no means of compressing the air and expelling it with sudden explosive force; he can simply inspire and expire forcibly and after exhausting efforts get rid of a little of the mucus. This desperate condition of affairs has led some surgeons to employ the dangerous and barbarous custom of introducing feathers and other irritants into the tube to stimulate the mucous membrane, which excites the patient and scatters the mucus both upwards and downwards. When somebody invents an appliance which will enable the patient to really cough through the tube, then tracheotomy will be placed upon a rational basis, and will stand some chance of becoming a useful operation. He thought tracheotomy had made a poor showing for its years of trial.

PROFESSOR G. C. PAOLI said that malignant diphtheria is a morbid poison, and that in epidemic cases there are very few recoveries. He stated that in such cases exudation takes place in the larynx or pharynx, and that an operation would only result in sending the patient more quickly to another world.

PROFESSOR W. E. QUINE said that he had operated twelve times for tracheotomy and had not had one recovery in diphtheritic cases. He knew he was not alone in an experience of unvarying failure in cases of this kind; and he knew some surgeons now regard tracheotomy with very little enthusiasm. It seemed to him unfair to place intubation of the larynx in contrast with tracheotomy upon the basis of the assumption that tracheotomy is always a *dernier resort*, that it is done when the patient is absolutely moribund, and that intubation is done under more favorable circumstances. This is not the fact. He said he was personally cognizant of two of Professor Waxham's cases in which the patients were *in extremis*, and in which death would undoubtedly have occurred in two or three hours had not relief been afforded. Surgeons rarely had occasion to perform tracheotomy under more discouraging circumstances.

DR. J. J. M. ANGEAR said he wished to call attention to a physiological and anatomical fact that had not been alluded to, viz.: that the arytenoid cartilages are not mature and that the chink of the glottis is held open by positive muscular action in small children, whereas in adults and older persons the arytenoid cartilages are mature and the chink is never closed. Dr. Angear said that a large number of children who suffocate will suffocate when there is no membrane present to cause suffocation, but simply some diseased condition that has interfered with the action of the delicate little muscles that draw back the arytenoid cartilages. When inflammatory action has interfered with these muscles drawing back the arytenoid cartilages, some mechanical interference like this tube will assist these muscles to keep the chink of the glottis open and let in air. He thought a large number of children who died of diphtheria did not choke to death, but died of poison in the system, and he did not think either the tube or tracheotomy, or any process, could save them. If there was interference with the opening of the chink of the glottis he had no doubt the introduction of the tube would save the life of the child.

PROF. J. S. KNOX said that the curse of tracheotomy is in the subsequent thoracic complications, either heart-clots or congestion and inflammation of the lungs, producing fatal results, and the reason probably is that tracheotomy is the final resort in cases of laryngeal obstruction. He thought that if tracheotomy were performed as early as intubation, there would be fifty per cent. of recoveries. The great advantage of intubation is that it can be performed early, and the early operation of intubation would no doubt save many a life that tracheotomy would not save if performed late. He thought that tracheotomy if performed as early as intubation would show as good results.

THE PRESIDENT said that he did not intend to say anything against the practice of intubation, but he did not believe it would take the place of tracheotomy. Intubation has had but a very short trial, and it is not yet time to pronounce it to be better than tracheotomy. The early experience of the President in tracheotomy had been almost the same as that of Dr. Waxham in intubation. In the first fifteen cases operated on but half of them recovered, and his later experience was better than that reported by Dr. Waxham, as within the last month he had had three cases of tracheotomy, all recoveries, while Dr. Waxham reports four cases of intubation, all fatal. So far as his own personal experience went, he thought tracheotomy had the advantage. He thought that if he should put a tube in a child's throat for the relief of laryngitis and the child died without his having performed tracheotomy, he should consider himself very much to blame. He had no doubt that cases of extreme diphtheritic laryngitis got well after tracheotomy; he had seen diphtheria of the pharynx and of the larynx recover after tracheotomy. Although he did not feel enthusiastic about intubation he thought it had a very good place and in many cases might be very useful, but that it could never supplant tracheotomy.

PROFESSOR WAXHAM said he had never found the tube occluded when it was removed. In one case when he introduced the tube a portion of the membrane was crowded down ahead of it, obstructing it entirely, and the tube was ejected and was then completely filled with membrane; the child recovered subsequently. On removing the tube on the fourth or fifth day he did not find it occluded.

DR. A. B. STRONG said, in conclusion, that he had had some experience with tracheotomy, having had twelve cases with but one recovery. He had no doubt that in cases of diphtheria the membrane could come up through the opening. The case which he had just reported was reported as spasmodic croup, and was not supposed to be one of false membrane, but he believed the child would have died without interference, and that belief was shared by Dr. Danforth, the attending physician. Dr. Strong said that he would hardly feel safe in leaving the tube in the trachea of a child without the thread. He agreed with the President that large pieces of membrane could not readily pass through such a long tube. In the case reported the tube was entirely free from membrane or pus when taken out.

---

CHICAGO MEDICAL SOCIETY.

*Stated Meeting, February 15th, 1886.* The President, C. T. PARKES, M.D., in the chair.

